# The Rid family member RutC of *Escherichia coli* is a 3-aminoacrylate deaminase

**DOI:** 10.1016/j.jbc.2021.100651

**Published:** 2021-04-09

**Authors:** Brandi A. Buckner, Ashley M. Lato, Shawn R. Campagna, Diana M. Downs

**Affiliations:** 1 Department of Microbiology, University of Georgia, Athens, Georgia, USA; 2 Department of Chemistry, University of Tennessee, Knoxville, Tennessee, USA

**Keywords:** rut, Rid superfamily, 3-aminoacrylate, deaminase, pyridoxal phosphate, 2-AA, 2-aminoacrylate, 3-AA, 3-aminoacrylate, CdsH, cysteine desulfhydrase, DMSO, dimethyl sulfoxide, ESI, electrospray ionization, HRMS, high-resolution mass spectrometry, LOX, lysyl oxidase, NCE, no-carbon E medium, NCN, no-carbon/nitrogen medium, rut, py*r*imidine *ut*ilization

## Abstract

The Rid protein family (PF14588, IPR006175) is divided into nine subfamilies, of which only the RidA subfamily has been characterized biochemically. RutC, the founding member of one subfamily, is encoded in the py*r*imidine *ut*ilization (*rut*) operon that encodes a pathway that allows *Escherichia coli* to use uracil as a sole nitrogen source. Results reported herein demonstrate that RutC has 3-aminoacrylate deaminase activity and facilitates one of the reactions previously presumed to occur spontaneously *in vivo*. RutC was active with several enamine–imine substrates, showing similarities and differences in substrate specificity with the canonical member of the Rid superfamily, *Salmonella enterica* RidA. Under standard laboratory conditions, a Rut pathway lacking RutC generates sufficient nitrogen from uracil for growth of *E. coli.* These results support a revised model of the Rut pathway and provide evidence that Rid proteins may modulate metabolic fitness, rather than catalyzing essential functions.

The Rid protein family (PF14588, IPR006175) has been split into nine subfamilies in the National Center for Biotechnology Information–conserved domain database (cd00448): RidA, Rid1-7, and RutC (1, 2). Members of the RidA subfamily are conserved across life and found in the majority of organisms in each domain. In contrast, members of the remaining subfamilies are found only in prokaryotes, primarily in the bacteria (1). Many prokaryotes encode multiple Rid proteins, often from different subfamilies. Despite the prevalence of these proteins, the vast majority of the Rid proteins have not been functionally characterized. The exception is the RidA subfamily. All RidA subfamily members tested have enamine deaminase activity (3, 4, 5, 6, 7). In *Salmonella enterica*, and the few other bacteria tested, hydrolysis of the reactive imine intermediate 2-aminoacrylate (2-AA) by RidA prevents metabolic damage that can occur when 2-AA accumulates *in vivo*. These studies convincingly support the hypothesis that 2-AA is the physiologically relevant substrate of RidA. Some members of the Rid1–3 subfamilies have imine–enamine deaminase activity when assayed with general amino acid oxidases, though the physiological relevance of these activities has not been determined (1, 8). Members of the Rid4–7 subfamilies lack the Arg105 residue (per *S. enterica* RidA numbering) that is critical for deaminase activity in RidA, and thus far, none have deaminase activity (1, 8).

*Escherichia coli* RutC is the founding member of the ninth Rid subfamily and encoded in the py*r*imidine *ut*ilization (*rut*) operon. The *rutABCDEFG* operon encodes enzymes required for the use of uracil (or uridine) as a nitrogen source and is divergently transcribed from a gene encoding helix–turn–helix protein (RutR) that is assumed to be an associated regulator (9, 10). A combination of *in vitro* and *in vivo* results (9, 10, 11, 12), including those herein, support the Rut pathway for uracil catabolism depicted in Figure 1. In this pathway, RutA/F catalyzes an uracil ring opening to produce ureidoacrylate (9, 11, 12). RutB hydrolyzes ureidoacrylate to result in the formation of carbamate and 3-aminoacrylate (3-AA), both of which were proposed to be spontaneously hydrolyzed to produce ammonia (9). The deaminated 3-AA (malonate semialdehyde) is then reduced by RutE (or YdfG) to form 3-hydroxy propionate. This final product of the pathway accumulates in the medium and is thought to be excreted as waste by the cell (9, 10).

**Figure 1:**
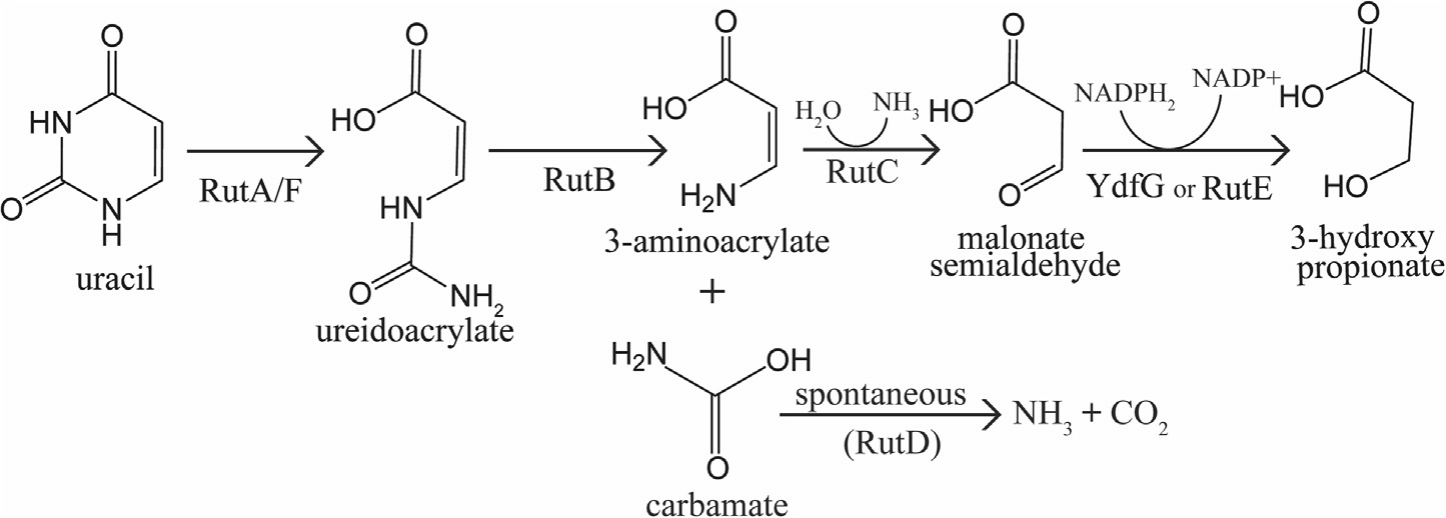
**The Rut pathway.** Uracil can be catabolized for use as a sole nitrogen source by the Rut pathway of *Escherichia coli* K12. The seven enzymes of the pathway are encoded by the *rut* operon, *rutABCDEFG*, the expression of which is predicted to be regulated by a divergently transcribed repressor encoded by *rutR* (9, 10). Once imported into the cell by the transporter RutG, uracil is converted to 2 mol of NH_3_, 1 mol of CO_2_, and 1 mol of 3-hydroxypropionate by RutA–F. YdfG can perform the reduction of malonate semialdehyde *in vitro*, but RutE is predicted to be the physiologically relevant enzyme. The deamination of 3-aminoacrylate can occur nonenzymatically or by catalysis by RutC. Carbamate is decarboxylated spontaneously, and the model suggests that RutD facilitates this reaction *in vivo.*

As described, the Rut pathway includes two critical steps that occur spontaneously in water. The presence of genes in the *rut* operon whose products have no assigned role raised the possibility that these steps might be catalyzed enzymatically *in vivo* by RutC or RutD, two enzymes that have not been biochemically characterized. In the first case, 3-AA undergoes a deaminating hydrolysis reaction, which is similar to the reaction attributed to RidA with 2-AA. RutC is a protein that has the arginine residue (Arg104) predictive of enamine deaminase activity of Rid family members (1, 8). This study was initiated to test the hypothesis that RutC was a 3-AA deaminase that catalyzed the deamination of 3-AA to malonate semialdehyde (9). Data herein show that RutC significantly increases the rate of 3-AA deamination over that achieved with solvent water. The further demonstration that *rutC* was not necessary for utilization of uridine *in vivo* was consistent with the spontaneous occurrence of the relevant reaction.

## Results and discussion

### RutC is a 3-AA deaminase

**Figure 2:**
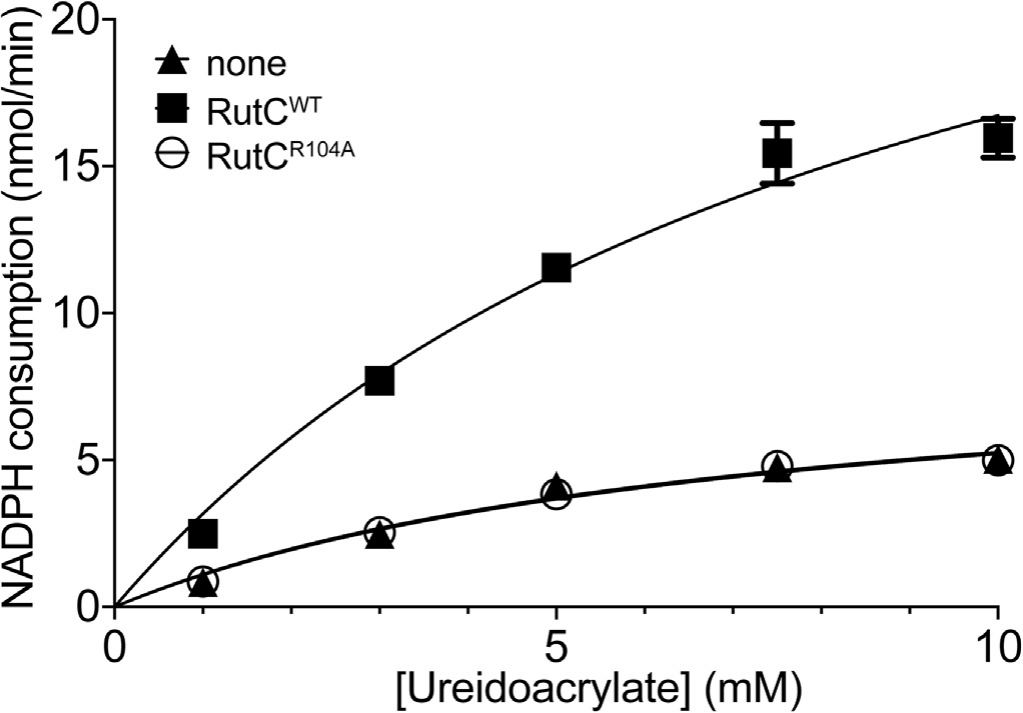
**RutC has 3-aminoacrylate deaminase activity *in vitro*.** A coupled-enzyme assay consisting of RutB (5 μM), YdfG (1 μM), and NADPH (0.8 mM) in 40 mM Tris–HCl buffer (pH 8.2) was performed at 23 °C in the presence and absence of RutC (3 μM). Reactions were initiated by addition of ureidoacrylate at varying concentrations. Using the change in absorbance at 340 nm from 200 to 230 s (absence of the change because of consumption of ureidoacrylate) and the extinction coefficient of NADPH (6.3 × 10^3^ M^−1^ cm^−1^), the rate of NADPH consumption in nanomole per minute was determined. The rate of NADPH consumption in the absence of RutC (*triangles*), the presence of RutC (*squares*), and the presence of RutC_R104A_ (*open circles*) is shown. Using Prism 7.0c, data were fit to the Michaelis–Menten equation, and the resulting curves are presented. Experiment was performed in technical duplicate; error bars represent standard deviation.

The hypothesized 3-AA deaminase activity of RutC was assessed using the coupled assay described in the Experimental procedures section. In this setup, RutB generated 3-AA *in situ* from chemically synthesized ureidoacrylate. *In situ* formation of 3-AA enabled the measurement of 3-AA deamination by RutC to yield malonate semialdehyde. Measurements of the NADPH-dependent rate of malonate semialdehyde reduction to 3-hydroxypropionate as a function of ureidoacrylate by *Ec*YdfG (malonate semialdehyde reductase; Enzyme Commission no.: 1.1.1.298) allowed us to determine apparent RutC kinetic parameters. The range of ureidoacrylate concentration that yielded a linear relationship to NADPH oxidation rate was between 1 and 4 mM (Fig. S1); we used 0.8 mM NADPH throughout our experiments. We determined in detail the rates of NADPH oxidation within the aforementioned range of ureidoacrylate to more reliably determine RutC kinetic parameters (Fig. 2; Table 1). Deamination of 3-AA by RutC contributed to an increased rate of NADPH consumption. Under optimal reaction conditions, and in the absence of RutC, ureidoacrylate was converted to malonate semialdehyde with an apparent *V*_max_ of 9 nmoles/min, reflecting the rate at which spontaneous deamination of 3-AA occurred. In the presence of RutC, the apparent *V*_max_ increased to 32 nmoles/min. Based on the assumption that RutB is turning over efficiently, apparent *K*_*m*_ and *k*_cat_ values were calculated for RutC (Table 1). No oxidation of NADPH was detected in the absence of either RutB or YdfG. Rid proteins lacking a key conserved arginine (residue 105 as per *S. enterica* numbering) have no detectable enamine deaminase activity (7, 8). Consistently, a RutC^R104A^ variant failed to increase in the rate of NADPH oxidation over that allowed by solvent water, indicating the RutC^R104A^ variant had no 3-AA deaminase activity (Fig. 2; Table 1).

**Table 1:** Kinetics of spontaneous and RutC-catalyzed deamination of 3-AA

Parameter	None	RutC	RutC^R104A^
*V*_max_	9.0 ± 1.0	31.9 ± 3.9	9.0 ± 0.9
*K*_*m*_ (mM)	7.1 ± 1.6	9.1 ± 1.9	7.3 ± 1.4
*k*_cat_	N/A	52	15

N/A, not applicable.

Assays included RutB (5 μM), YdfG (1 μM), and the indicated RutC protein (3 μM when present) in Tris–HCl (40 mM, pH 8.2). Further details of the assay are included in the Experimental procedures section. *V*_max_ is reported as nmoles of NADPH consumed min^−1^, *k*_cat_ is reported as min^−1^ and was not applicable for the spontaneous reaction. Standard error is reported.

### RutC and RidA have distinct substrate specificity *in vitro*

**Figure 3:**
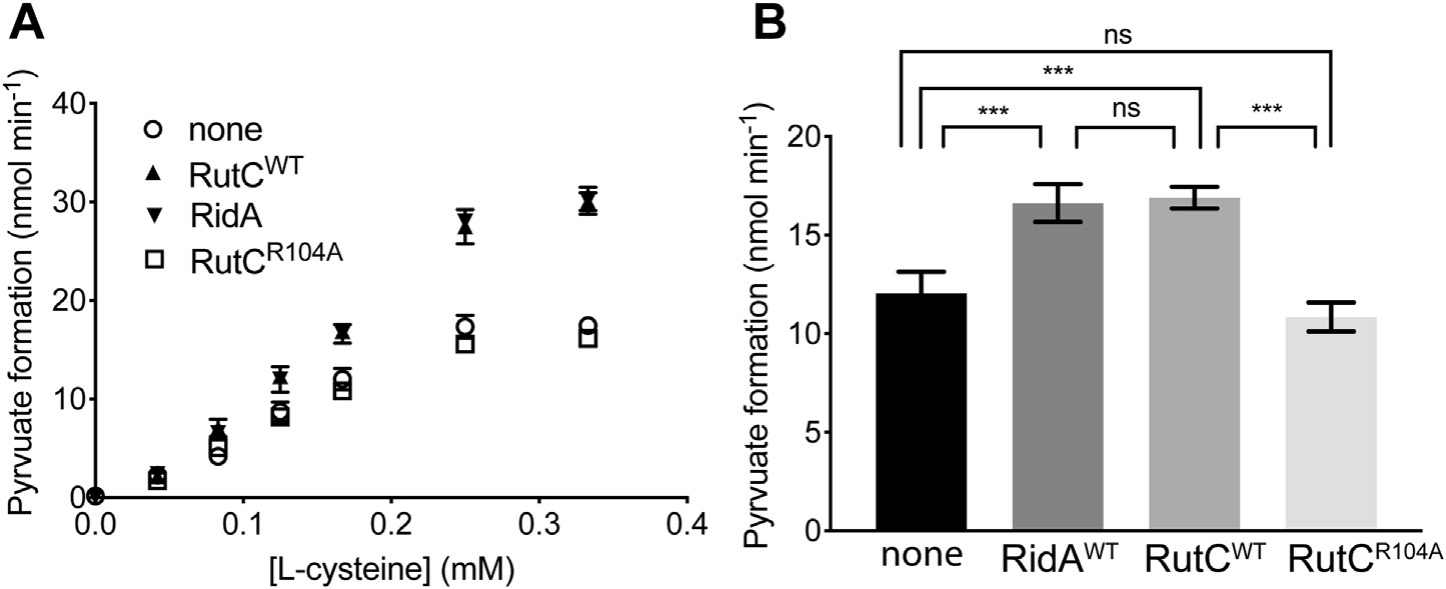
**RutC has 2-aminoacrylate deaminase activity *in vitro*.** Reactions containing 0.3 μM RidA or RutC, lactate dehydrogenase (5 U), CdsH (0.2 μM), pyridoxal 5′-phosphate (30 μM), and NADH (0.25 mM) in Tris–HCl buffer (100 mM, pH 8) were initiated with varying concentrations of l-cysteine (l-cys). The oxidation of NADH was monitored by the absorbance of NADH at 340 nm. The rate of pyruvate formation was calculated using the molar absorptivity of NADH (6220 M^−1^ cm^−1^) and the change in absorbance at 340 nm from 20 to 50 s. This experiment was performed in technical triplicate; error bars represent standard deviation. *A*, the rate of pyruvate formation (nmoles min^−1^) as function of l-cys concentration (millimolar). *B*, rate of pyruvate formation (nmoles min^−1^) at 0.17 mM l-cys. Statistical significance was determined by one-way ANOVA followed by Tukey's post hoc test in Prism 7.0c (GraphPad); three asterisks = *p* < 0.001.

**Figure 4:**
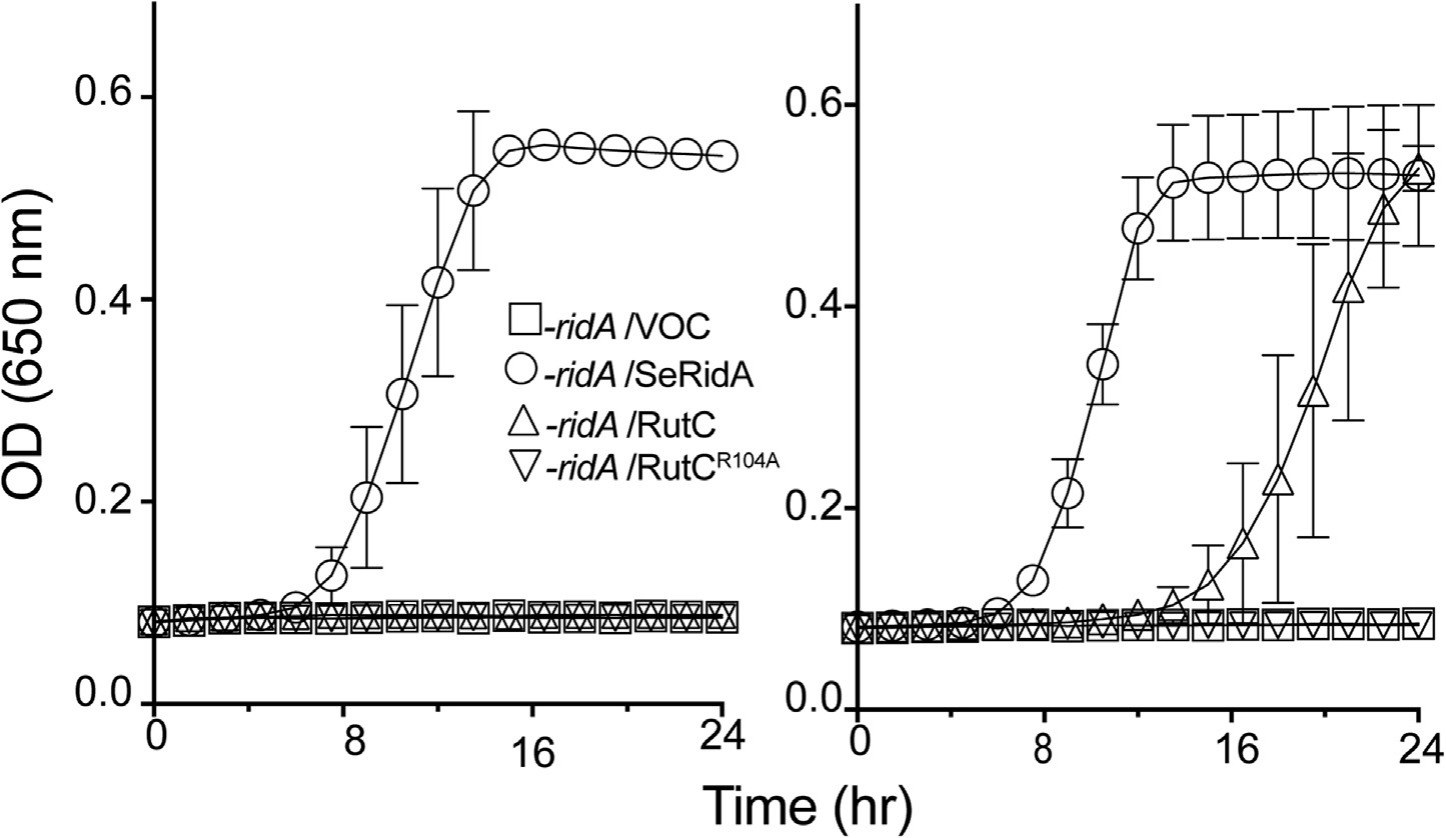
***Escherichia coli rut*C complements a *Salmonella enterica ridA* mutant.** In minimal medium supplemented with serine, a *S. enterica rid*A mutant carrying the empty l-arabinose-inducible vector pCV1 (*open squares*, DM15419) has a growth defect. pCV1 constructs were expressed *in trans* to test for complementation without (panel *A*) and with (panel *B*) induction by L(+)-arabinose (0.2%). The *ridA* strains carried plasmids expressing *Se*RidA (*circles*, DM15421), RutC (*triangle*, DM17321), or RutC^R104A^ (*inverted triangles*, DM17322). This experiment was performed in biological triplicate; error bars represent standard deviation. All symbols have error bars, but in some cases, the bars are smaller than the symbol.

3-AA differs from the physiologically relevant substrate of RidA, namely 2-AA, by the position of the amino group. The overall similarity of the molecules suggested there could be functional overlap between members of the RidA subfamily and RutC. RidA did not deaminate 3-AA, emphasizing the unique activity of RutC (data not shown). In contrast, RutC efficiently deaminated 2-AA generated *in situ* by the cysteine desulfhydrase (CdsH) enzyme acting on cysteine (Fig. 3). Numerous Rid proteins outside the RidA subfamily have activity with 2-AA *in vitro* under standard conditions (8). Members of the RidA subfamily restore growth to a *S. enterica ridA* mutant with the weak expression allowed by the uninduced pBAD promoter. In contrast, when *rutC* is provided *in trans*, growth of the *ridA* mutant was restored only when expression was induced. Furthermore, while the culture reached a final density equal to that of a wildtype strain, it had a significantly extended lag time. These results suggested that RutC could deaminate 2-AA *in vivo*, albeit less efficiently than RidA. Finally, expression of the allele encoding a RutC^R104A^ variant failed to restore growth of the *ridA* mutant with or without induction (Fig. 4). Thus, RutC joined the group of Rid proteins proficient at 2-AA deamination *in vitro* but only poorly able to suppress phenotypes of a *S. enterica ridA* mutant *in vivo* (8). This result emphasizes that the *in vitro* analysis of 2-AA deaminase activity as currently done cannot be used as a definitive measure of physiological relevance.

**Figure 5:**
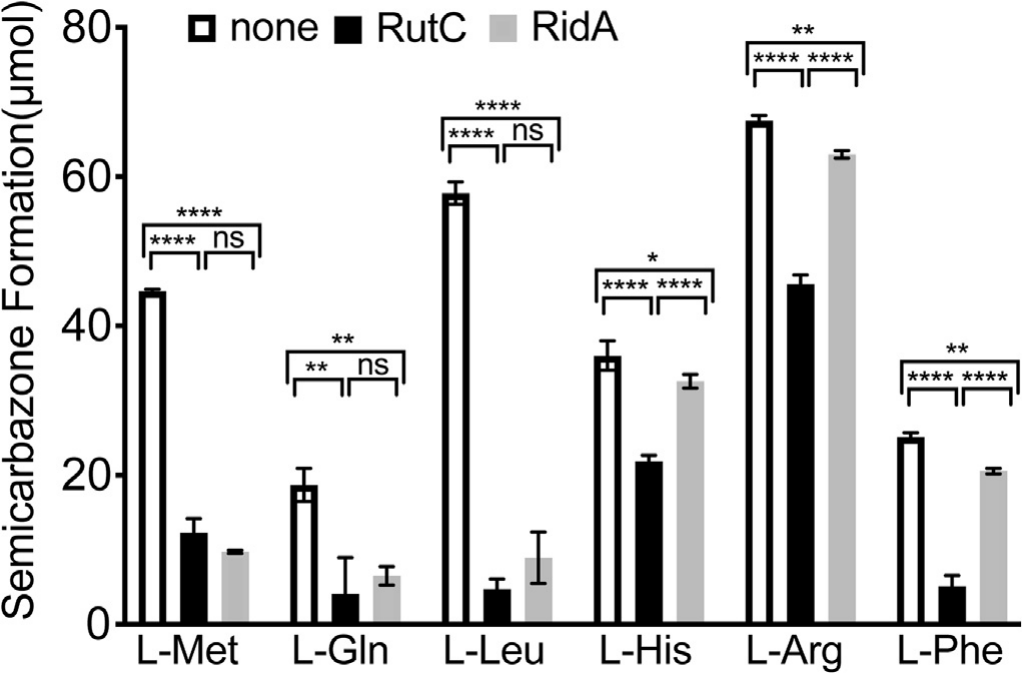
**RidA and RutC have distinct imino–aminoacid substrate specificity.** Assays containing 1 μg bovine liver catalase, 1 μg l-amino acid oxidase (LOX), and 10 mM neutralized semicarbazide in 50 mM potassium pyrophosphate (pH 8.7) were performed at 23 °C in the presence or the absence of 10 μM of RidA or RutC. Reactions were initiated with addition of 10 mM l-amino acid. The rate of semicarbazone formation was measured by monitoring absorbance at 248 nm. Using the change in absorbance at 240 nm from 0 to 10 min and the extinction coefficient of semicarbazone (10,300 M^−1^ cm^−1^), the total amount of semicarbazone formed was calculated. Experiments were performed in technical triplicate; error bars indicate standard deviation. One-way ANOVA followed by Tukey's post hoc test was used to determine statistical significance; one *asterisk* indicates an adjusted *p* value between 0.01 and 0.05; two *asterisks* indicate an adjusted *p* value between 0.01 and 0.001; three *asterisks* indicate an adjusted *p* value between 0.001 and 0.0001; and four *asterisks* indicate an adjusted *p* < 0.0001.

Flavin adenine dinucleotide–dependent amino acid oxidase enzymes (l-amino acid oxidase [LOX]) have been used to explore a diversity of substrates used by Rid proteins beyond those with attributed metabolic relevance (1, 5, 8, 13). LOX enzymes oxidize amino acids to their corresponding iminoamino acids, which can be deaminated by solution water to generate the respective ketoacid (14). An imine will react with semicarbazide present in the reaction mix to form a semicarbazone that absorbs at 248 nm. It follows that if the rate of semicarbazone formation decreases in the presence of a Rid protein, the respective imine is a substrate of that protein. Six amino acids that were efficient substrates of LOX (8) were used to probe RutC substrate specificity. Using these amino acids as substrates, the rate of semicarbazone formation was determined when RutC, RidA, or no Rid protein was provided in the reaction (Fig. 5). RidA showed activity primarily on the imino derivatives of methionine and leucine. These results were consistent with those previously reported (8). In contrast, RutC had a broader substrate specificity with significant activity on each of the six iminoamino acids (Fig. 5). The most significant difference between the RidA and RutC proteins was noted when histidine, arginine, or phenylalanine was substrates for LOX. While none of these iminoamino acids have defined roles in the metabolism of *E. coli* or *S. enterica*, the results suggested differences in the active site configuration of the two proteins from different subfamilies of the Rid superfamily.

### Neither RutC nor RutD is required to catabolize uridine as sole nitrogen source *in vivo*

**Figure 6:**
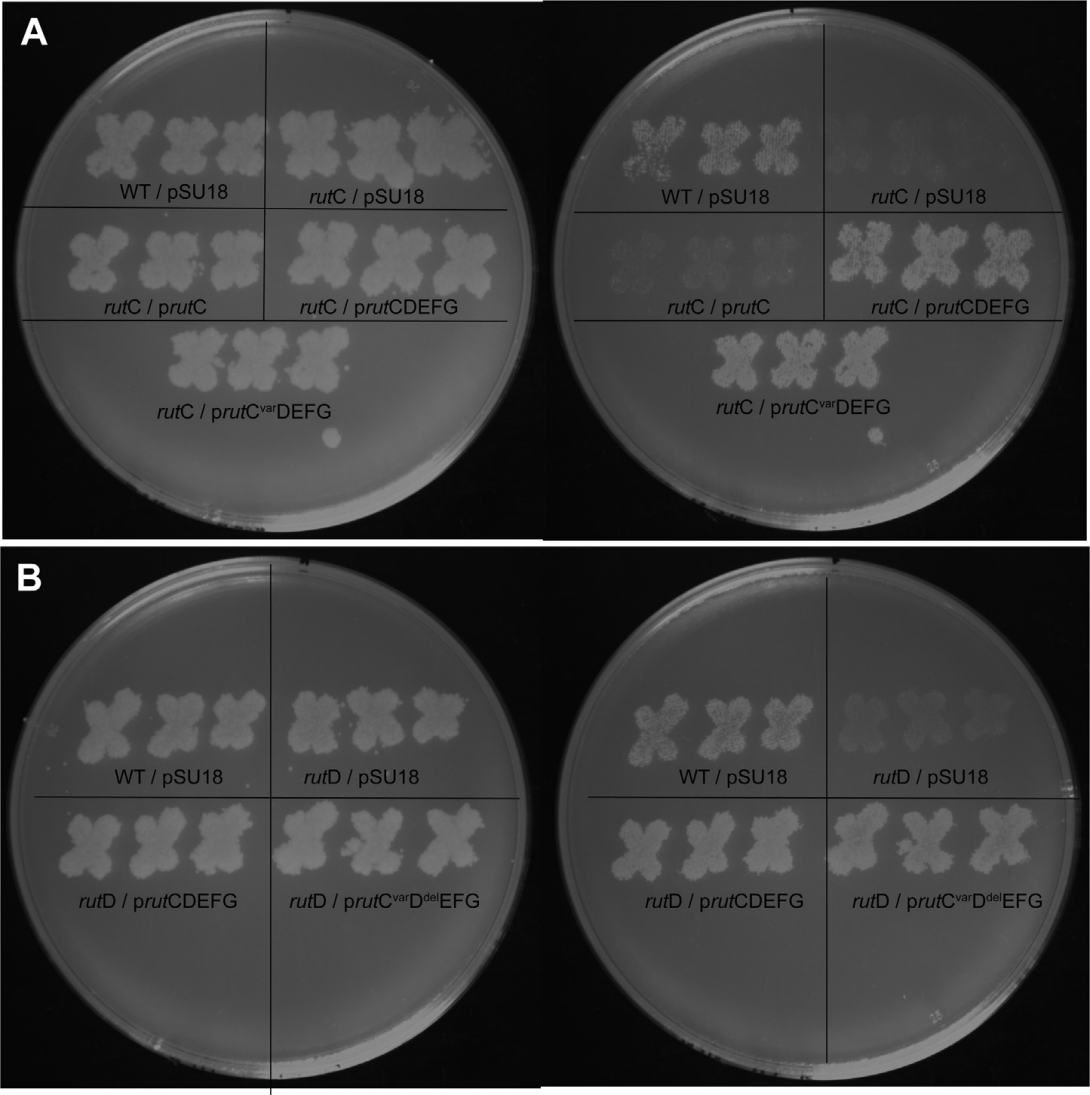
***Escherichia coli rut*C and *rut*D are not required for growth with uridine as a sole nitrogen source.** At room temperature, wildtype *Escherichia coli* K12 (NCM3722) grows on no-carbon E medium uridine media (where ammonium and uridine are available nitrogen sources; *left hand plates*) and no-carbon/nitrogen (NCN) uridine media (where uridine is the sole nitrogen source; *right hand plates*). pSU18 constructs were expressed *in trans* to probe essentiality of *rut*C mutant strain (NCM4105) and *rut*D mutant strain (NCM4861). The plasmid p*rut*C (pDM1663) encodes RutC, and plasmid p*rut*CDEFG (pDM1667) encodes RutC–G of the *rut* operon. The plasmid p*rut*C_var_DEFG (pDM1668) carries a *rut*C allele encoding an inactive RutC^R104A^ variant. The plasmid pDM1661 (p*rut*C^var^D^del^EFG) encodes a *rut*C allele encoding RutC^R104A^ and a *rut*D allele encoding a truncated RutD^ΔV16-L250^. *A*, growth of *rutC* mutants with indicated plasmids on no-carbon E uridine medium (*left*) and NCN uridine medium (*right*). *B*, growth of *rutD* mutants on minimal media NCE uridine medium (*left*) and NCN uridine medium (*right*).

The 3-AA deaminase reaction attributed to RutC can occur in the absence of enzyme with solvent water, raising the question whether this enzyme would be required for the Rut pathway *in vivo*. The Δ*rut*C strain NCM4105 carrying an empty cloning vector did not grow at room temperature (23 °C) with uridine (5 mM) as sole nitrogen, as reported (9) (Fig. 6*A*). Unexpectedly, expression of *rutC in trans* did not restore growth of this strain, suggesting that the lesion in *rutC* was polar on downstream gene(s) required for *rut* pathway function. Consistent with this scenario, a plasmid carrying the complete *rutCDEFG* operon restored growth of Δ*rutC* strain with uridine as sole nitrogen source. Importantly, when the *rutC* gene within the operon was mutated to encode the inactive RutC^R104A^ variant, the resulting plasmid also restored growth of the Δ*rutC* strain on uridine. These results demonstrated that *rutC* was not essential for Rut pathway function under the laboratory conditions used.

The role of RutD in the catabolic pathway was unclear from past work, and a plausible model is that this enzyme could facilitate the hydrolysis of carbamate (Fig. 1). A Δ*rutD* strain NCM4861 carrying an empty vector was unable to use uridine, as previously reported (9). A plasmid expressing the complete *rutCDEFG* operon allowed growth of the Δ*rutD* mutant. Surprisingly, a plasmid that encoded an inactive variant of RutC (RutC^R104A^) and a truncated variant of RutD restored growth (Fig. 6*B*). These data demonstrated that the Δ*rut*D strain could not catabolize uridine because of polarity of the lesion on one or more of the downstream *rut* genes. Thus, the results were consistent with a model in which RutD (in addition to RutC) facilitates a reaction than can occur spontaneously.

## Conclusions

### RutC deaminates 3-AA

RutC is the founding member of a ninth Rid subfamily and located within the *rutABCDEFG* operon in *E. coli*, which encodes enzymes required for the use of uracil (or uridine) as a nitrogen source (9, 10). Data herein demonstrated that RutC catalyzes the deamination of 3-AA, a proposed intermediate in the Rut pathway. The assignment of an enzymatic function to RutC is an important correction to the literature (9, 15).

RutC is similar to biochemically characterized members of the RidA subfamily in that it accelerates a deamination reaction that is catalyzed by water. Significantly, the substrate of RutC is not acted upon by RidA, despite the fact that these enzymes share other substrates *in vitro*, including the physiological substrate 2-AA. As expected, the active site arginine that has been associated with the deaminase activity of other Rid proteins is present in RutC, and a variant lacking it (RutC^R104A^) is inactive. Additional studies, both with RutC and other Rid proteins, are needed to define structural components responsible for the substrate specificity of RutC and facilitate the ability to categorize Rid family members and predict their physiological roles.

### RutC is an enzyme, but the conditions where its activity is required remain an open question

Despite demonstrable 3-AA deaminase activity of the purified protein *in vitro*, RutC was not required for *E. coli* to utilize uridine as nitrogen source under the conditions tested. Other enzymes of the pathway were required for growth with uridine as nitrogen source under these same conditions. This result suggests that the rate of spontaneous 3-AA hydrolysis is sufficient to generate enough nitrogen for robust growth under laboratory conditions. The prevalence of *rutC* in bacterial species that have a Rut pathway suggests that the enzyme may provide a competitive advantage under as-yet-unknown conditions. We consider it likely that the laboratory conditions used do not detect a fitness defect that would be relevant in the natural environment. Taken together, the current data suggest that the action of RutC (and potentially RutD) might result in higher nitrogen levels that could be critical for competition in a specific environmental niche. This scenario would be consistent with the emerging model in which the unifying feature of Rid superfamily members is that they accelerate reactions that occur nonenzymatically *in vivo* (4). This model further suggests that the RidA subfamily prevents metabolic damage caused by a reactive intermediate (16), whereas members of other subfamilies increase the efficiency of metabolic processes that might otherwise be constrained by the rate of spontaneous hydrolysis in the cell (17). The presence of Rid proteins in operons encoding catabolic pathways throughout bacteria intimates that multiple additional catalytic activities will be uncovered for members of this widespread protein superfamily.

## Experimental procedures

### Bacterial strains, media, and chemicals

Strains used in this work, and their source, are indicated in Table 2. Lysogeny broth (tryptone [10 g/l], yeast extract [5 g/l], and NaCl [5 g/l]), super broth (tryptone [32 g/l], yeast extract [20 g/l], NaCl [5 g/l], and 1 ml of 1 M NaOH per liter), and nutrient broth (nutrient broth [8 g/l] and NaCl [5 g/l]) were used as rich media for both growth studies and protein overexpression. No-carbon E (NCE) medium (1× NCE, 1 mM MgSO4) or no-carbon/nitrogen (NCN) medium (1× NCN, 1 mM MgSO_4_) containing uridine (5 mM), glycerol (0.4%), and agar (15 g/l) were used as minimal media in growth studies of *E. coli* (18). NCE medium (1× NCE, 1 mM MgSO4) containing 0.1× trace minerals, glucose (11 mM), and ampicillin (15 μg/ml) was used as minimal medium in *S. enterica* growth studies. Serine (5 mM) was added to the medium as indicated. Chloramphenicol (20 μg/ml), ampicillin (150 μg/ml), and kanamycin (50 μg/ml) were included in rich medium to maintain plasmids.

**Table 2:** Strains, plasmids, and primers

Strain	Genotype	Source
NCM3722	*E. coli* K12	(9)
DM13778	K12/pSU18	This study
NCM4105	K12 Δ*rutC*	(9)
DM17047	K12 *ΔrutC*/Pdm1666	This study
DM13779	K12 *ΔrutC*/Pdm1667	This study
DM13743	K12 *ΔrutC*/Pdm1668	This study
NCM4861	K12 *ΔrutD*	(9)
DM13746	Δ*rutD*/pSU18	This study
DM13747	Δ*rutD*/pDM1667	This study
DM17308	Δ*rutD*/pDM1661	This study
DM15418	*S. enterica* LT2/pCV1	Laboratory collection
DM15419	*S. enterica* LT2 *ridA3*::MudJ/pCV1	Laboratory collection
DM15421	*S. enterica* LT2 *ridA3*::MudJ/DM1439	Laboratory collection
DM17321	*S. enterica* LT2 *ridA3*::MudJ/pDM1663	This study
DM17322	*S. enterica* LT2 *ridA3*::MudJ/pDM1664	This study
BL21-AI		Laboratory collection
DH5α		Laboratory collection
DM16701	K12 strain AG1/pCA24N-ydfG	(23)
Plasmid	Description	Source
pSU18	Expression vector, Cm^R^	(24)
pDM1666	pSU18-*rutC*	Laboratory collection
pDM1667	pSU18-*rutCDEFG*	Laboratory collection
pDM1668	pSU18-*rutC*^(310–312,CGA→GCA)^*DEFGG*	Laboratory collection
pDM1661	pSU18-*rutC*^(310–312,CGA→GCA)^*D*^(Δ46–750)^*EFGG*	This study
pCV1	Arabinose-inducible expression vector, Amp^R^	(21)
pDM1439	pCV1-*ridA* (*Salmonella enterica*)	Laboratory collection
pDM1663	pCV1-*rutC*	This study
pDM1664	pCV1-*rutC*^(310–312;CGA→GCA)^; encodes RutC^R104A^	This study
BspQI-Modified pET28b	Expression vector for *C*-terminal His_6_, KanR	(22)
pDM1405	Modified pET28b-*rutC*	Laboratory collection
pDM1638	Modified Pet28b-*rutC*^(310–312;CGA→GCA)^; encodes RutC^R104A^	This study
pCA24N-*ydf*G	Expression vector encoding *E. coli ydfG*	(23)
Primer	Sequence	
PR42	5′-NNGCTCTTCNATGCCAAAATCCGTAATTATTCCC-3′	
PR43	5′-NNGCTCTTCNGTGCTTGGCGATATGCG-3′	
PR1513	5′-NNGCTCTTCNTTCATGCCAAAATCCGTAATTATTCCC-3′	
PR1513	5′-NNGCTCTTCNTTATCACTTGGCGATATGCGC-3′	
PR1511	5′-TTACTCAACGGGCTTGCCAG-3′	
PR1512	5′-TACGGGCGCATCAGCATAAGG-3′	

Strains are *Escherichia coli* unless noted otherwise.

### 2H-1,3(3H)-oxazine-2,6-dione (oxauracil) synthesis

Chemical reagents were purchased commercially and used without further purification. Synthesis of oxauracil was performed using previously described methods (19). Briefly, trimethylsilyl azide (1.5 ml, 11 mmol) was added dropwise to a cooled solution (0 °C) of maleic anhydride (1.0 g, 10 mmol) in methylene chloride (10 ml) under an inert nitrogen atmosphere. After stirring for 2 h, the mixture was warmed to room temperature and left to stir overnight. Absolute ethanol (∼40 ml) was added dropwise to the reaction until precipitate formation ceased. The solid product was collected *via* filtration and recrystallized from ethanol, yielding 890 mg (77%) of off-white crystalline product. High-resolution mass spectrometry (HRMS) (electrospray ionization [ESI]) *m/z*: [M + H]^+^ calculated for C_4_H_4_NO_3_^+^, 114.0186; found 114.0188. ^1^H NMR (dimethyl sulfoxide [DMSO]-d_6_, 500 MHz): δ 5.60 to 5.61 (d, 1H), 7.64 to 7.65 (d, 1H), 11.52 (s, 1H). ^13^C NMR (DMSO-d_6_, 500 MHz): δ 94.7, 146.6, 148.8, and 159.8 (Fig. S2).

### Ureidoacrylate synthesis

Ureidoacrylate was synthesized as described elsewhere (9). In a round bottom flask, 3 ml of 4 M ammonium hydroxide was added to 100 mg 3-oxauracil on ice and stirred until dissolved. The reaction was left at room temperature for 12 h before adding 1 ml of 1 M NaOH. The mixture was lyophilized, and the resulting solid was extracted by adding 1 ml of methanol. The methanol fraction was dried using Eppendorf Vacufuge concentrator. NMR analysis confirmed the formation of ureidoacrylate (Fig. S2). HRMS (ESI) *m/z*: (M − H)^−^ calculated for C_4_H_5_N_2_O_3_^−^, 129.0306; found 129.0302. ^1^H NMR (DMSO-d_6_, 500 MHz): δ 4.54 to 4.57 (d, 1H), δ 5.43 (s, 1H), δ 6.33 (s, 2H), δ 6.72 to 6.81 (t, 1H), and δ 11.08 to 11.11 (d, 1H).

### NMR and mass spectrometry

^1^H NMR and ^13^C NMR spectra were recorded on a Varian Inova 500 MHz instrument (Varian, Inc) in solutions of (CD_3_)_2_SO. HRMS was performed using an Exactive Plus Orbitrap mass spectrometer (Thermo Scientific) with an ESI source.

### Assay for 3-AA deaminase activity

A coupled-enzyme assay (final volume of 200 μl) containing RutB (5 μM), RutC (3 μM), YdfG (1 μM), NADPH (0.8 mM), and ureidoacrylate in Trizma base buffer (40 mM, pH 8.2 at 23 °C) was used to assay RutC activity. Each experiment included technical duplicates and was performed multiple times. The change in absorbance of NADPH at 340 nm was monitored in a quartz microplate with Spectramax Plus 384 microplate reader (Molecular Devices) and Softmax Pro 6.2 software (Molecular Devices). The absorbance of ureidoacrylate at 340 nm was controlled for by carrying out blank reactions without NADPH and subtracting these rates from the data obtained in the full reaction. The assay was initiated by the addition of varying amounts of ureidoacrylate. The amount of NADPH consumed was determined by the decreased absorbance at 340 nm using the molar absorptivity of NADPH (6220 M^−1^ cm^−1^). NADPH (0.8 mM) was added (9), and its consumption over time was determined (Fig. S1). Rates of NADPH oxidation *versus* ureidoacrylate concentration were graphed in Prism, version 7 (GraphPad) to define apparent kinetic parameters.

### Assay for 2-AA deaminase activity

A coupled-enzyme assay (final volume of 300 μl) consisting of CdsH (0.19 μM), lactate dehydrogenase (5 U), NADH (0.25 mM), pyridoxal-5-phosphate (30 μM), RidA (0.3 μM) or RutC (0.3 μM), and l-cysteine (l-cys) in Trizma base buffer (100 mM, pH 8.0 at 23 °C) was used to determine 2-AA deaminase activity (20). Assay was performed in technical triplicate in a quartz microplate and initiated by addition of varying concentrations of l-cys. Using Spectramax Plus 384 microplate reader and Softmax Pro 6.2 software, the rate of pyruvate formation was quantified by measuring the change in absorbance of NADH at 340 nm (6200 M^−1^ cm^−1^) from 20 to 50 s. Prism, version 7, was used to graph data and determine statistical significance by one-way ANOVA followed by Tukey's post hoc test.

### Assay for iminoacid deaminase activity

A coupled-enzyme assay (final volume of 100 μl) consisting of bovine liver catalase (1 μg), l-amino acid oxidase (1 μg), neutralized semicarbazide (10 mM), RidA (10 μM) or RutC (10 μM), and various l-amino acids (10 mM) in potassium pyrophosphate buffer (50 mM, pH 8.7 at 23 °C) was performed to determine iminoacid deaminase activity (8). Reactions were initiated with addition of l-amino acids, and the change in absorbance at 248 nm was monitored using a Spectramax Plus 384 microplate reader and Softmax Pro 6.2 software. The assay was performed in technical triplicate in a quartz plate. Using the molar absorptivity of semicarbazone at 248 nm (10,300 M^−1^ cm^−1^) and the change in absorbance at 248 nm from 0 to 10 min, the rate of semicarbazone formation was calculated. Prism, version 7, was used to plot data and determine statistical significance by one-way ANOVA followed by Tukey's post hoc test.

### Growth studies

Isolated colonies of *E. coli* were patched in biological triplicate on LB agar plates containing chloramphenicol and incubated at 37 °C for 14 to 16 h. Patched plates were replica printed onto NCN medium and NCE agar plates containing uridine (0.5 mM), MgSO_4_ (1 mM), and glycerol (0.4%). Replica-printed plates were incubated at room temperature (23 °C) for 2 days.

*S. enterica* growth studies were performed in biological triplicate. Inoculum was prepared by incubating 1 ml nutrient broth cultures containing ampicillin at 37 °C with shaking for 16 to 18 h. Cells were pelleted and resuspended in 1 ml NaCl solution (8.5 g/l). Resuspended cells were used to inoculate NCE media in a microwell plate (2% inoculum). The plate was incubated in a Biotek EL808 ultra microplate reader set to 37 °C with constant shaking. Growth was quantified by monitoring the absorbance at 650 nm.

### Molecular biology

Wildtype *rutC* and the allele encoding RutC^R104A^ (nucleotides 310–312 were changed from CGA to GCA) were PCR amplified from pDM1677 and pDM1668, respectively, using primers PR1513 and PR1514 for cloning into vector pCV1 (21) and primers PR42 and PR43 for cloning into pET28b-SAPKOCH. PCR products and vectors were digested with BspQI and ligated together using technique described by Galloway *et al*. (22). The partial in-frame deletion of *rutD* found in pDM1661 was generated using primers PR1511 and PR1512 to PCR amplify the regions of pDM16568 upstream and downstream of the portion of *rutD* targeted for deletion (nucleotides 46–750). The blunt ends of the PCR product were ligated with plasmid to generate pDM1661. In all cases, ligation mixtures were transformed into DH5α, and transformants were selected for the appropriate antibiotic resistance. Plasmids were verified for sequence by Eurofins Genomics.

### Protein overexpression and purification

#### YdfG

An LB chloramphenicol culture of BL21 carrying pCA24N-ydfG (1.5 ml) was used to inoculate 1.5 l of LB containing chloramphenicol. Cells were grown at 37 °C with shaking (150 RPM). When an absorbance at 600 nm reached ∼0.5, cells were induced by adding IPTG to 100 μM. Cultures were grown for 17 h at 15 °C (150 RPM) and harvested at 4000 RPM for 10 min at 4 °C. The resulting cell pellet was stored at –80 °C until use.

Cells were thawed and resuspended (5 ml/g) in bind buffer (potassium phosphate at pH 7.4 [20 mM], NaCl [300 mM], and glycerol [10%]) containing PMSF (1 mM), DNase (20 U/ml), and lysozyme (1 mg/ml). Cells were lysed using OneShot cell disruptor at 20 psi. Lysate was cleared at 40,000*g*, 4 °C for 45 min. Cleared supernatant was loaded onto Ni-agarose column and washed with bind buffer and then wash buffer (potassium phosphate at pH 7.4 [20 mM], imidazole [25 mM], NaCl [300 mM], and glycerol [10%]). Protein was eluted with potassium phosphate buffer (20 mM, pH 7.4) containing imidazole (250 mM), NaCl (300 mM), and glycerol (10%). Elution fractions were collected and visualized on SDS-PAGE gel. Fractions containing target protein were combined and dialyzed in potassium phosphate buffer (20 mM, pH 7.4) containing NaCl (300 mM) and glycerol (10%). Buffers were changed three times reducing NaCl concentration stepwise reaching a final buffer of potassium phosphate (20 mM, pH 7.4) containing NaCl (100 mM) and glycerol (10%). Protein was flash frozen in liquid nitrogen and stored at −80 °C. Protein was >95% pure (Fig. S3).

#### *RutC*

One and a half liter of super broth containing kanamycin was inoculated with BL21/pET28b-rutC, and cultures were incubated at 37 °C 150 RPM until an absorbance at 350 nm reached ∼0.6 to 0.8. Expression was induced with the addition of l-arabinose (0.2% [w/v]) and IPTG (100 μM), and the cultures were incubated at 30 °C 150 RPM for 16 to 18 h. Cells were harvested at 4000*g* for 20 min (4 °C) and resuspended (5 g/ml) in bind buffer (potassium phosphate [50 mM, pH 8.0], NaCl [100 mM], glycerol [10%], imidazole [20 mM]) with lysozyme (1 mg/ml), DNase (0.125 mg/ml), and PMSF (1 mM). Cells were lysed at 20 psi using OneShot cell disruptor. Lysate was cleared at 40,000*g* for 45 min at 4 °C. Cleared lysate was filtered with a 0.45 uM polyethersulfone filter. Filtered lysate was applied to a 5 ml His-trap column and washed with 4% elution buffer (potassium phosphate [50 mM, pH 8.0], NaCl [100 mM], glycerol [10%], and imidazole [500 mM]). The protein was eluted in a gradient from 4% to 100% elution buffer. Fractions of eluted protein were visualized by SDS-PAGE and Coomassie blue staining. Fractions containing target protein were combined and placed into dialysis. Dialysis buffers were changed four times with the final storage buffer containing potassium phosphate buffer (50 mM, pH 8.0) and glycerol (10%). Proteins were flash frozen in liquid nitrogen and stored at −80 °C. Protein was >95% pure (Fig. S3).

## Data availability

The data that support the findings of this study are within the article or the supplementary material of this article, and additional information is available upon request from the authors.

## Supporting information

This article contains supporting information.

## Conflict of interest

The authors declare that they have no conflicts of interest with the contents of this article.
