# Supplementary material for: The Rid family member RutC of Escherichia coli is a 3-aminoacrylate deaminase
Source: J Biol Chem. 2021 Apr 9;296:100651. doi: 10.1016/j.jbc.2021.100651 (PMC8113886; doi:10.1016/j.jbc.2021.100651)
Supplement: Figures S1 to S3 [file mmc1.pdf]

SUPPORTING INFORMATION

The Rid family member RutC of *Escherichia coli* is a 3-aminoacrylate deaminase

Brandi A. Buckner[1], Ashley M. Lato[2], Shawn R. Campagna[2] and Diana M. Downs[1]*

[1]Department of Microbiology
University of Georgia, Athens, GA 30602

[2]Department of Chemistry
University of Tennessee, Knoxville, TN 37996

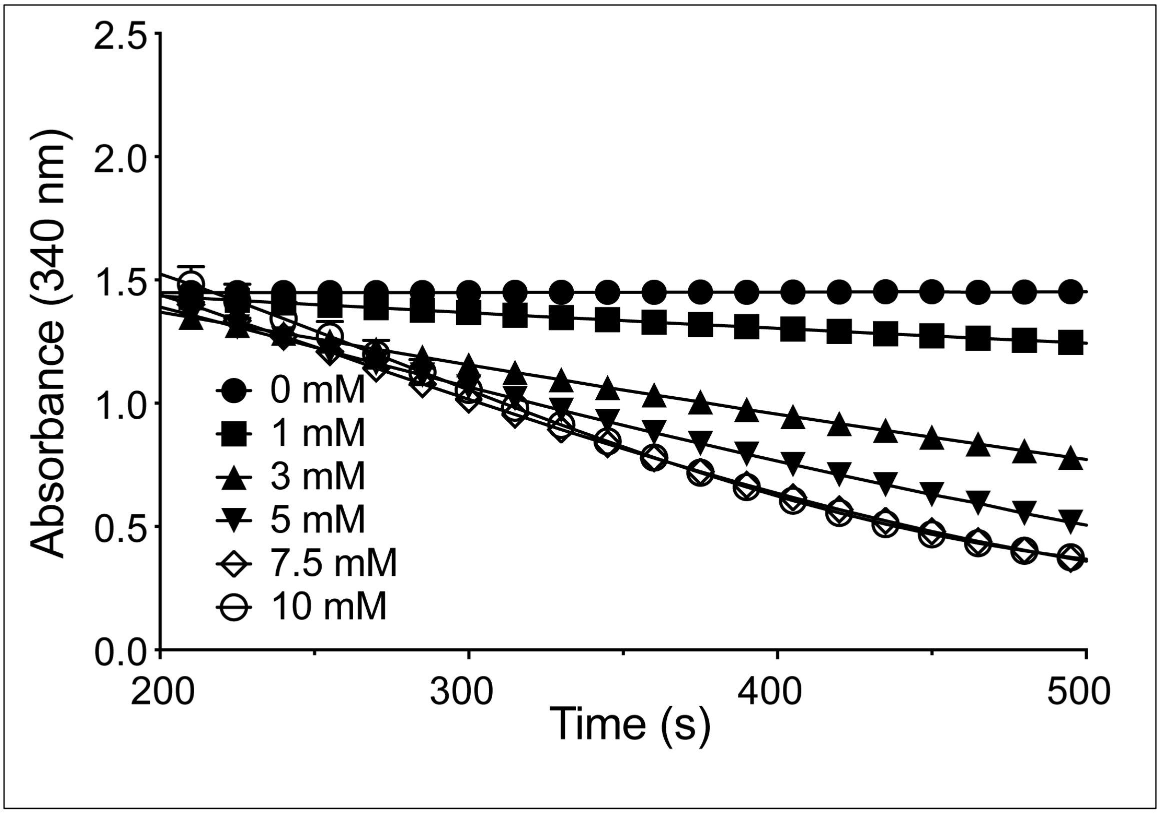

Figure S1. **Rate of 3-AA deamination by RutC**.

Raw data from coupled-enzyme assays consisting of RutB (5 µM), YdfG (1 µM), NADPH (0.8 mM), RutC (3 µM) and different concentrations of ureidoacrylate in 40 mM Tris•HCl buffer (pH 8.2) performed at room temperature (23 °C). Decrease in absorbance of 340 nm includes contributions from consumption of ureidoarcylate by RutB and consumption of NADPH by YdfG.

Figure S2. Confirmation of synthesis of (A) oxauracil and (B) ureidoacrylate by ^{1}H NMR and ^{13}C NMR as noted.

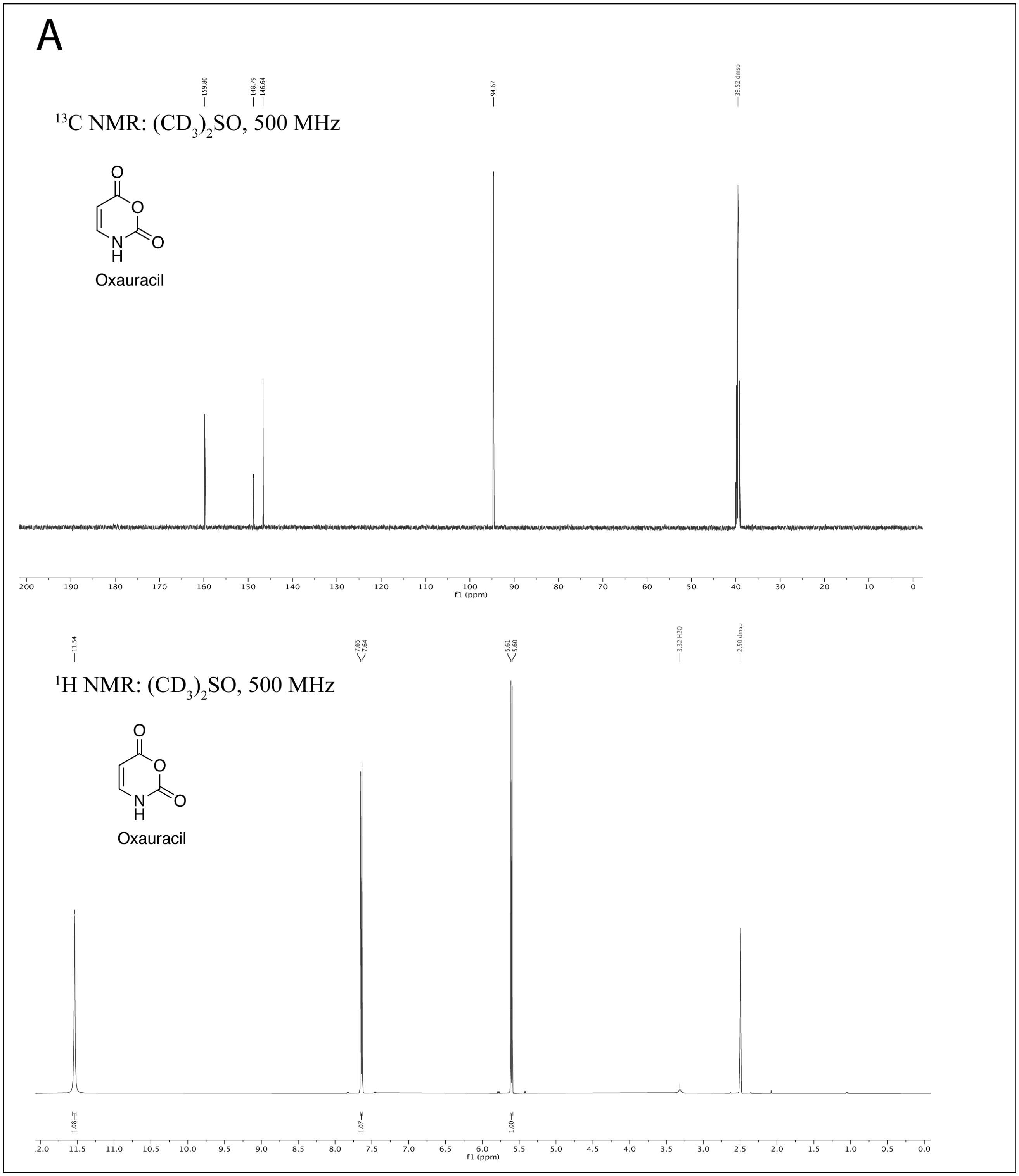

^{1}H NMR (DMSO-d_6, 500 MHz): δ 5.60-5.61 (d, 1H), 7.64-7.65 (d, 1H), 11.52 (s, 1H). ^{13}C NMR (DMSO-d_6, 500 MHz): δ 94.7, 146.6, 148.8, 159.8.

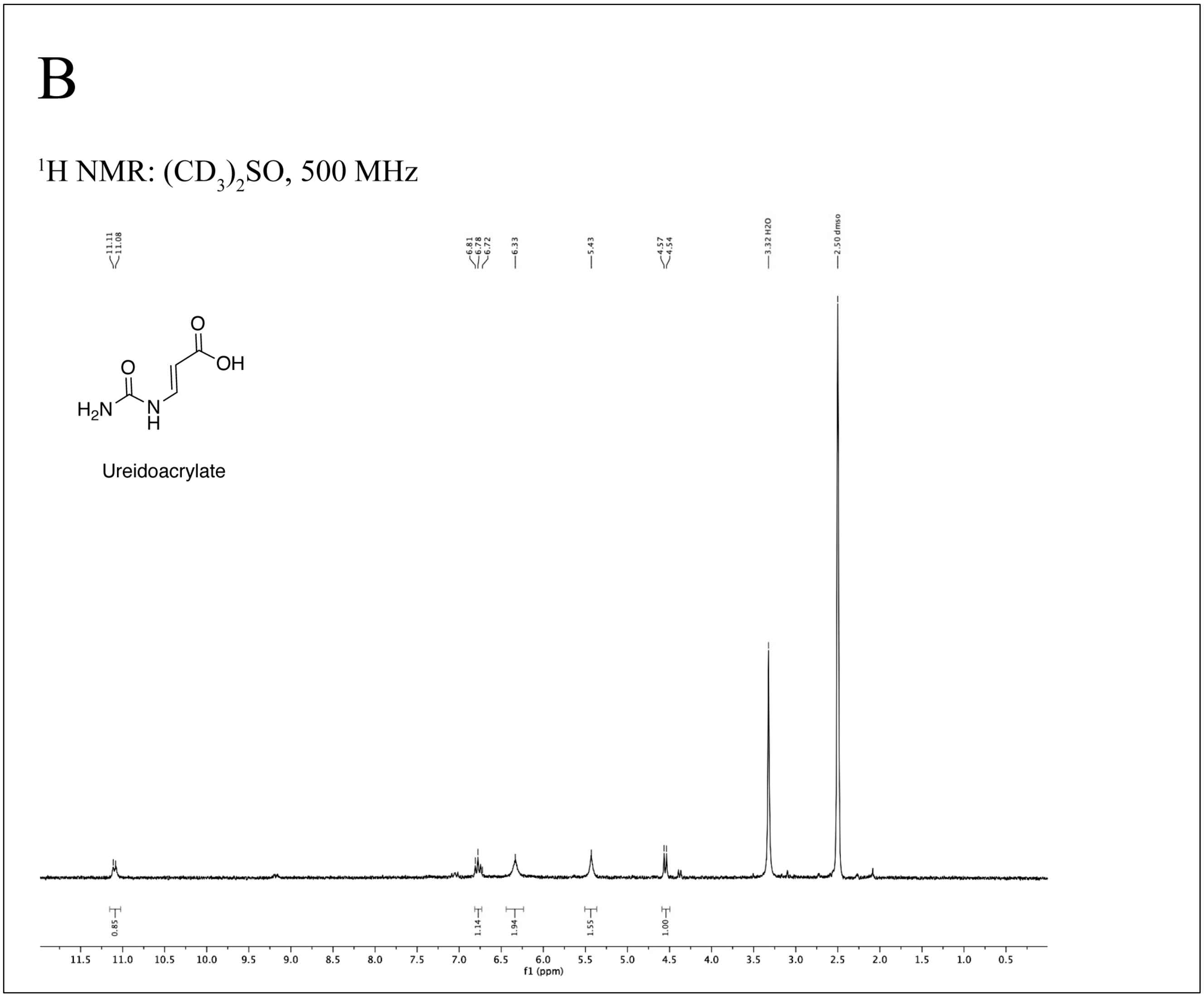

^{1}H NMR (DMSO-d_6, 500 MHz): δ 4.54-4.57 (d, 1H), δ 5.43 (s, 1H), δ 6.33 (s, 2H), δ 6.72-6.81 (t, 1H), δ 11.08-11.11 (d, 1H).

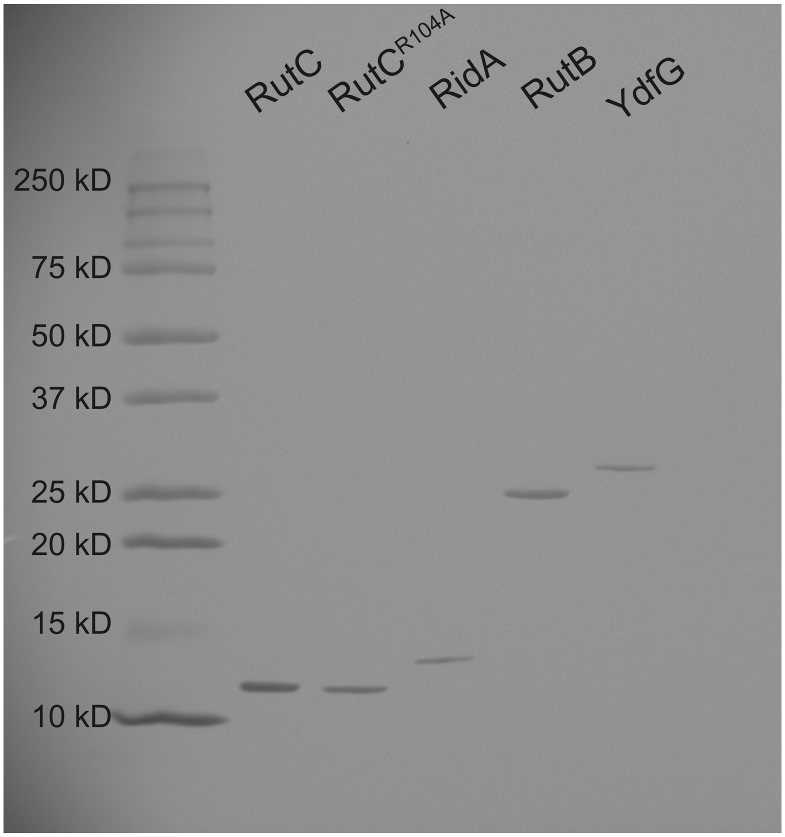

Figure S3. Purified Proteins. Two µg of each purified His-tagged protein visualized by SDS-PAGE and Coomassie blue staining. Proteins are RutC from *E. coli*, RutC^{R104A}, RidA from *Salmonella enterica*, RutB from *E. coli*, YdfG from *E. coli*, as indicated.
